# The prognostic significance of circulating plasma cells in newly diagnosed multiple myeloma patients

**DOI:** 10.3389/fonc.2023.1266868

**Published:** 2023-09-20

**Authors:** Weiqin Yao, Haifei Yang, Hongying You, Jingjing Shang, Yingying Zhai, Zhi Yan, Shuang Yan, Xiaolan Shi, Ying Yao, Jing Wang, Panfeng Wang, Yun Xu, Song Jin, Lingzhi Yan, Depei Wu, Chengcheng Fu

**Affiliations:** ^1^ National Clinical Research Center for Hematologic Diseases, Jiangsu Institute of Hematology, The First Affiliated Hospital of Soochow University, Suzhou, China; ^2^ Key Laboratory of Thrombosis and Hemostasis of Ministry of Health, The First Affiliated Hospital of Soochow University, Suzhou, China; ^3^ Suzhou Hongci, Hematology Hospital, The First Affiliated Hospital of Soochow University, Suzhou, China

**Keywords:** multiple myeloma, flow cytometry, circulating plasma cells, cytogenetic risk factors, prognosis

## Abstract

**Objective:**

Multiple myeloma (MM) is a highly characteristic tumor that is influenced by numerous factors that determine its prognosis. Studies indicate that the presence of circulating plasma cells (cPCs) is a detrimental factor that significantly impacts the prognosis of patients with MM.

**Methods:**

This study retrospectively analyzed the prognostic value of cPCs quantified by 10-color flow cytometry in 145 newly diagnosed MM (NDMM) cases in the First Affiliated Hospital of Soochow University from November 2018 to February 2021. The study was approved by the Ethics Committee of the hospital (2021 No. 93).

**Results:**

Of the 145 patients, 99 (68.2%) were detected cPCs. Through receiver operating characteristics (ROC) analysis, an optimal threshold of 0.165% was identified as a predictor for overall survival (OS). The median progression-free survival (PFS) was 33 months in patients with cPCs ≥0.165%, whereas those with cPCs <0.165% had a PFS of <33 months (p=0.001). The median OS was not reached for two groups; the 3-year OS for patients with cPCs ≥0.165% was 71% compared with 87% for those with cPCs <0.165% (p=0.003). In transplant patients, cPCs ≥0.165% also predicted worse prognosis. Similarly, when considering cytogenetic risk factors in conjunction with cPC levels, comparable results were obtained. To evaluate whether the Revised International Staging System (R-ISS) groups could be further stratified based on different prognostic factors related to cPCs, our study revealed similar median PFS and OS rates in R-ISS II stage patients with cPCs ≥0.165% compared to those in the III stage (p=0.659 and 0.249, respectively).

**Conclusion:**

This study demonstrates that a high ratio of cPCs serves as a reliable indicator for predicting a poorer prognosis in MM cases. Furthermore, incorporating the R-ISS system and cytogenetic risk factors alongside the level of cPCs enhances the accuracy of prognostic predictions for patients with MM.

## Background

Multiple myeloma (MM) ranked as the second most prevalent cancer among all hematological malignant tumors (1). According to relevant statistical results, there were 16,500 new cases and 10,300 deaths of MM in China in 2016. The incidence rate was 1.03 per 100,000 population in 2016 (2). The pathogenesis of MM is closely related to the proliferation of plasma cell. Consequently, information pertaining to plasma cells plays a crucial role in supporting the diagnosis and prognosis of this disease. The treatment technology for MM has significantly improved in recent years; however, despite these advancements, MM remains an incurable disease. Therefore, accurately identifying high-risk factors and implementing risk stratification can offer valuable support in enhancing treatment precision and prognosis for patients with MM.

The prognosis in MM is associated with factors such as tumor burden, biology of the tumor, and its sensitivity to treatment (3, 4). The evaluation of tumor burden in MM has been carried out using the Durie–Salmon staging system (DSS), which was established in 1975 (5), and the International Staging System (ISS), which was established in 2005 (6), whereas the molecular subtype of the disease and the presence of secondary cytogenetic abnormalities, such as del(17p), gain(1q), or del(1p), provide the best reflection of the disease’s biology (7, 8). To establish a comprehensive prognostic index that aids in clinical treatment and facilitates the comparison of clinical trial data, the Revised International Staging System (R-ISS) was introduced in 2016, combining tumor burden (ISS) with disease biology (9). In 2018, Mayo updated the mSMART 3.0 Prognostic Stratification System, which provides information on the likelihood of double-hit myeloma (10). However, cases characterized by the t(4;14) translocation and abnormal gain(1q) experience a notable decrease in survival rates (11). These findings highlight the limitations of current risk classification models in the context of advanced treatment and emphasize the importance of stratifying multiple myeloma based on comprehensive prognostic factors (12).

As it is known, bone marrow plasma cells examination is a golden standard for the evaluation of the tumor burden and an indicator to assess the prognosis and response for MM patients. Several studies utilizing various methods suggested that there were small numbers of plasma cells in the peripheral blood, namely, circulating plasma cells (CPCs), and demonstrated that cPCs are a high-risk factor for plasma cell disease, including monoclonal gammopathy of undetermined significance (MGUS), smoldering multiple myeloma (SMM), and MM. Patients with high levels of cPCs are significantly more prone to developing dominant diseases (13, 14). Comparative analysis shows that there are obvious differences in genetics between plasma cell leukemia (PCL) and MM. PCL is defined as more than two circulating plasma cell per liter × 10^9^; the proportion of the cloned plasma cell in blood is higher than 20%. However, the study has also found that patients with more than 5% of circulating plasma cell also had poor prognosis, suggesting the need for a revision in the disease definition (15).

This study was to verify if the cPCs can be applied as a biomarker to enhance the prognostic performance of R-ISS system for newly diagnosed multiple myeloma patients.

## Methods

### Patients

A total of 145 NDMM patients were included at the First Affiliated Hospital of Soochow University between November 2018 and February 2021. All patients were enrolled in a registered clinical trial and treated with VRD (Bortezomib with lenalidomide and dexamethasone) in combination with autologous stem cell transplantation or VRD treatment for eight cycles. The study was approved by the ethics committee of the Hospital of Soochow University (No. 93, 2021). Prior to initiating therapy, peripheral blood samples from these patients were analyzed using a 10-color flow cytometry technique. The diagnosis and staging of the patients were determined based on the International Myeloma Working Group (IMWG) criteria (16, 17). Patients with PCL were excluded from the study.

### Flow cytometry

Ethylenediaminetetraacetic acidperipheral blood (EDTA-PB) samples were collected from 145 MM patients when diagnosed, and 10-color flow cytometry was evaluated within 24 h after collection. Samples were labeled with antibodies CD138-APC/CD38-APC750/CD45-KO/CD19-ECD/CD56-PC7/CD27-PB/CD81-APC700/CD117-PC5 (Beckman Coulter, Beijing, China) and cytoplasmic Kappa and Lambda immunoglobulin light chains (Dako, Shanghai, China). Beckman Coulter Navios flow cytometry was used for analysis. The gating strategy was applied with the level of CD38 and CD138 to identify all plasma cells. The cPCs were identified based on Kappa and that chains restricted expression. At least 50,000 nucleated cells were analyzed for each tube. The percentage of CPC was expressed as CPC/total nucleated cells from the blood. CPC negativity was defined as the absence of CPC in the blood with a limit of detection of <1×10^−4^, while CPC positivity was defined as the level of CPC higher than this threshold.

### Characteristics and cytogenetics

The different variables were examined for hemoglobin, albumin, serum calcium (CA), creatinine, lactate dehydrogenase (LDH), β2 microglobulin (β2-MG), M protein quantification, cPCs, and BMPC proportion. Fluorescent *in situ* hybridization (FISH) was performed for 145 patients who had BM samples available at initial diagnosis. Seven chromosomal abnormalities, including t(11;14), t(4; 14), t(14; 16), 1q21 amplification, 13q14 deletion, Rb1 deletion, and P53 deletion, were detected by interphase fluorescence *in situ* hybridization (iFISH) on CD138-sorted plasma cells. High-risk cytogenetic abnormalities include t(4; 14), t(14;16), and P53 deletion.

### Statistical analysis

The endpoints were OS and PFS. OS was measured from the date of diagnosis until the time of death. PFS was calculated from the day of diagnosis until the occurrence of death, disease progression, or relapse. Statistical analysis was conducted based on Statistical 22.0. Chi-square test and F-tests were applied to check tabular data and the t-test to analyze continuous data between sub-groups. The Kaplan–Meier method was used to create PFS and OS curves, and log-rank test was applied to check the curves. ROC curve was applied to obtain the optimal threshold of cPCs that predicted for worse PFS and OS. Finally, a multivariable analysis was conducted based on the Cox method to evaluate the influence of different factors on PFS and OS.

## Results

A total of 145 NDMM cases were included in the study, and their corresponding peripheral blood samples were analyzed using 10-color flow cytometry at the time of diagnosis. The patient characteristics, disease features, and treatment methods are presented in **Table 1**. The median age of the patients was 58 years ranging from 31 to 73, and 57.9% of the patients were men. The distribution of disease stages was as follows: 26 patients (17.9%) were classified as ISS stage I, 63 patients (43.4%) as stage II, and 56 patients (38.6%) as stage III. Regarding R-ISS staging, 21 patients (14.5%) were categorized as stage I, 97 patients (66.9%) as stage II, and 27 patients (18.6%) as stage III. A total of 36 (24.8%) cases had high-risk cytogenetics, and 47 (32.4%) cases had renal insufficiency (creatinine clearance rate <40ml/min × 1.73m^2^). Of the patients, 73.8% underwent autologous stem cell transplantation (ASCT) following induction treatment.

**Table 1:** Disease and clinical characteristics of the 145 NDMM patients.

Variables	All patients (N=145)
Male (N, %)	84(57.9%)
Age	58(31, 73)
ISS stage (N, %) stage I stage II stage III	26(17.9%) 63(43.4%) 56(38.6%)
R-ISS stage (N, %) stage I stage II stage III	21(14.5%) 97(66.9%) 27(18.6%)
Hemoglobin (g/L)	90.9 ± 24.8
Platelets (×10^9^/L)	171.2 ± 66.5
Albumin (g/L)	33.6 ± 6.9
Globulin (g/L)	44.5(12.5, 125.8)
Lactate ehydrogenase (LDH) (U/L)	157.6(59.9, 611.9)
Creatinine clearance rate (CCR) ml/(min×1.73m^2^) <40 ≥40	47(32.4%) 98(67.6%)
Calcium (mmol/L)	2.2(1.8, 3.6)
β2-microglobulin (mg/L)	4.0(0.86, 64.2)
Circulating plasma cells (cPCs,≥0.165%) Positive Negative	99(68.2%) 46(33.8%)
M-protein (g/L)	20.4(0.21, 104.5)
Bone marrow plasma cell (BMPC) (%)	28.0(10.0, 95.5%)
FISH (N,%) High risk Standard risk	36(24.8%) 109(75.2%)
Autologous stem cell transplantation (ASCT) (N, %) Yes No	107(73.8%) 38(26.2%)

Patients were enrolled in the study between November 2018 and February 2021, and they were followed up until October 2021. The median duration of follow-up was 22 months, ranging from 1 to 38 months. Of the 145 patients, 99 (68.2%) had cPCs detected. Using the ROC method, it was determined that the optimal cutoff value for predicting the highest risk of OS was approximately 1,650 cPCs per 1,000,000 events (0.165%). This cutoff yielded an area under the curve (AUC) of 0.698, with a sensitivity of 0.647 and a specificity of 0.727. The patients who had cPCs ≥0.165% showed lower hemoglobin and platelet levels (p<0.001 and 0.001) and higher CA and BMPC levels (p=0.025 and <0.001, respectively). Patients with cPCs ≥0.165% were found to have later stages according to ISS and R-ISS (p<0.001). There were more renal insufficiency and high-risk cytogenetic patients with cPCs ≥0.165% (p=0.004 and 0.001) (**Table 2**).

**Table 2:** Comparison of clinical characteristics of two groups divided by cPCs.

Variables	CPCs ≥0.165% (N=42)	CPCs <0.165% (N=103)	p-value
Male (N, %)	24(57.1%)	60(58.3%)	0.902
Age	58(45, 72)	58(31, 73)	0.443
ISS stage (N, %) stage I stage II stage III	2(4.8%) 13(31.0%) 27(64.2%)	24(23.3%) 50(48.5%) 29(28.2%)	<0.001
R-ISS stage (N, %) stage I stage II stage III	1(2.4%) 21(50.0%) 20(47.6%)	20(19.4%) 76(73.8%) 7(6.8%)	<0.001
Hemoglobin (g/L)	77.2 ± 23.1	96.5 ± 23.3	<0.001
Platelet (×10^9^)	142.8 ± 62.5	182.8 ± 62.4	0.001
Albumin (g/L)	33.3 ± 7.3	33.8 ± 6.9	0.699
Globulin (g/L)	49.8(12.5, 125.8)	43.7(14, 112.2)	0.856
LDH (u/L)	166.6(90.2, 611.9)	154.5(59.9, 458.2)	0.160
CCR: ml/(min×1.73m^2^) <40 ≥40	21(50.0%) 21(50.0%)	26(25.2%) 77(74.8%)	0.004
Calcium (mmol/L)	2.3(1.8, 3.6)	2.2(1.8, 3.2)	0.025
β_2_-microglobulin (mg/L)	7.3(2.3, 64.2)	3.1(0.86, 39.0)	<0.001
M-protein (g/L)	25.7(0.23, 104.5)	18.9(0.21, 86.5)	0.318
BMPC(%)	55.5(10.0, 95.0%)	22.0(10.0, 95.5)	<0.001
FISH(N,%) High risk Standard risk	18(42.9%) 24(57.1%)	18(17.5%) 85(82.5%)	0.001

**Figure 1:**
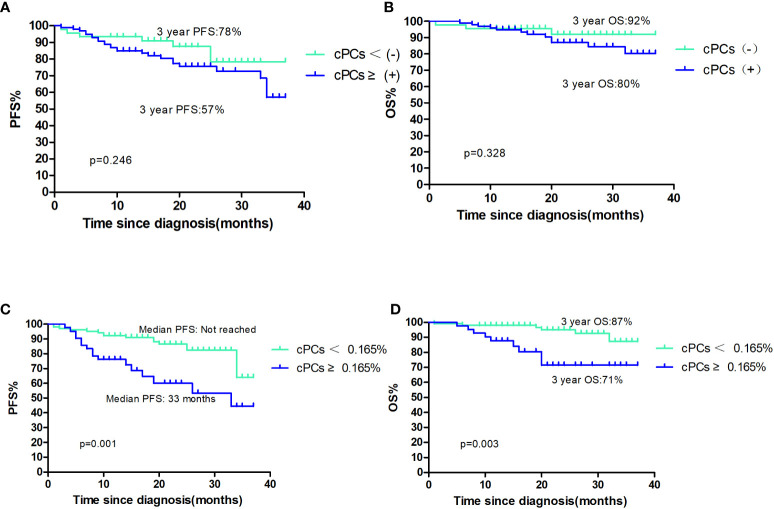
**(A, B)** The PFS and OS of patients with cPCS(+)or cPCs(−); **(C, D)** the PFS and OS of patients with cPCS ≥0.165% or cPCs<0.165%.

A total of 15 (10.7%) patients had died, and 32 (21.3%) patients had died or relapsed during the follow-up period. The median PFS and OS were not reached; the 3-year PFS and OS for these cases was 59% and 83%, respectively. The median PFS and OS were not reached for patients with or without cPCs; 3-year PFS were 57% and 78%, respectively (p=0.246, **Figure 1A**), 3-year OS were 80% and 92%, respectively (p=0.328, **Figure 1B**). Among the patients with cPCs, those with cPCs ≥0.165% had a median PFS of 33 months, whereas those with cPCs <0.165% had a median PFS not reached (p=0.001, **Figure 1C**). The median OS was not reached in either group; the 3-year OS rates were 71% for patients with cPCs ≥0.165% and 87% for patients with cPCs <0.165% (p=0.003, **Figure 1D**).

**Figure 2:**
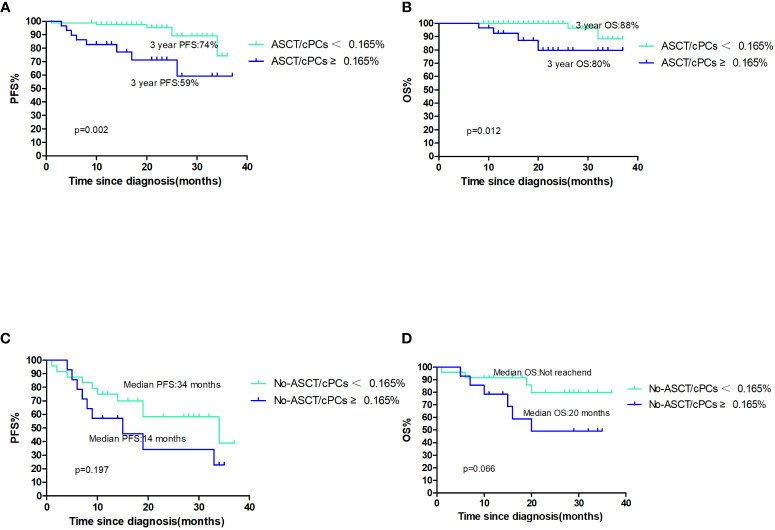
**(A, B)** The PFS and OS of patients with ASCT/cPCS(+)or ASCT/cPCs(−); **(C, D)** the PFS and OS of patients with No-ASCT/cPCS ≥0.165% or No/ASCT cPCs<0.165%.

Out of the total patient population, 107 patients underwent ASCT. Among them, 29 patients (27.1%) had circulating plasma cells (cPCs) ≥0.165% at the time of diagnosis, while 78 patients (72.9%) had cPCs <0.165%. The median PFS and OS were not reached in both cPCs ≥0.165% and cPCs <0.165% groups. The 3-year PFS was 59% in patients with cPCs ≥0.165% and 74% in patients with cPCs <0.165% (p=0.002, **Figure 2A**). The 3-year OS rate was 80% in patients with cPCs ≥0.165% and 88% in patients with cPCs <0.165% (p=0.012, **Figure 2B**). In other 38 patients who had not received ASCT, the median PFS and OS in patients with cPCs ≥0.165% were only 14 months and 20 months, while these were much shorter than that in patients with cPCs <0.165% of 34 months and not reached (p=0.197 and 0.066, **Figures 2C, D**). As a conclusion, no matter in ASCT or non-ASCT patients, patients with cPCs ≥0.165% all had poor clinical outcomes significantly (p<0.05).

We conducted a regression analysis to examine the impact of multiple variables (**Table 3**) on PFS and OS. The variables assessed included age, ISS stage, renal insufficiency, and high-risk cytogenetics on FISH results with either t(4;14), t(14,16), or del(17p), LDH levels, the presence of ≥0.165% cPCs, and ASCT. In the univariate model, the presence of ≥0.165% cPCs, LDH levels, ISS stage, high-risk cytogenetics, and ASCT were found to affect PFS. The presence of ≥0.165% cPCs, LDH levels, and ASCT were found to significantly affect OS. Further analysis revealed that, in this model, only the presence of ≥0.165% cPCs, high-risk cytogenetics, LDH levels, and ASCT were clearly associated with PFS. Similarly, the presence of ≥0.165% cPCs, LDH levels, and ASCT were significantly related to OS.

**Table 3:** Univariable and Multivariable analysis of factors predicting PFS and OS in NDMM patients.

Variable	PFS				OS			
	Univariable		Multivariable		Univariable		Multivariable	
	p	HR (95%CI)	p	HR (95%CI)	p	HR (95%CI)	p	HR (95%CI)
Age (≥65)	0.188	1.687 (0.774, 3.674)	–	–	0.131	2.307 (0.780, 6.825)	0.146	2.652 (0.711, 9.893)–
ISS stage II/III	0.049	7.387 (1.008, 54.1125)	0.125	4.946 (0.642, 38.096)	0.266	–	–	–
CCR ≥40	0.056	1.979 (0.981, 3.989)	0.407	0.715 (0.323, 1.580)	0.117	–	–	–
High risk	0.025	2.246 (1.109, 4.551)	0.434	1.331 (0.651, 2.721)	0.042	2.877 (1.041, 7.951)	0.738	1.210 (0.397, 3.682)
LDH	<0.001	1.005 (1.002, 1.008)	0.001	1.005 (1.002, 1.007)	<0.001	1.008 (1.004, 1.011)	<0.001	1.008 (1.004, 10.13)
cPCs ≥0.165%	0.001	3.040 (1.512, 6.111)	0.041	2.118 (1.032, 4.349)	0.003	4.319 (1.532, 12.176)	0.036	3.315 (1.079, 10.182)
ASCT	<0.001	0.216 (0.106, 0.440)	<0.001	0.209 (0.091, 0.448)	0.001	0.175 (0.060, 0.513)	0.004	0.186 (0.060, 0.580)

**Figure 3:**
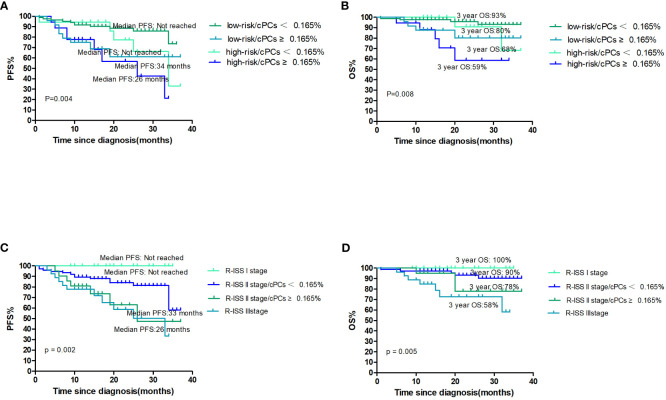
**(A, B)** The PFS and OS according to cytogenetics and cPCs; **(C, D)** the PFS and OS according to R-ISS stage and cPCs.

The cytogenetics on FISH results with either t(4;14), t(14,16), or del(17p), in conjunction with the level of cPCs, was used to evaluate outcomes of NDMM patients. Among the patients classified as having standard-risk cytogenetics, the median PFS and OS were not reached in either group. The 3-year PFS rate was 61% in patients with cPCs ≥0.165% and 73% in patients with cPCs <0.165% (p=0.012). The 3-year OS rate was 80% in patients with cPCs ≥0.165% and 93% in patients with cPCs <0.165% (p=0.040). On the other hand, in the high-risk cytogenetics group, the median PFS was 26 months for patients with cPCs ≥0.165% and 34 months for patients with cPCs <0.165% (p=0.164). The median OS was not reached for either group; the 3-year OS rate was 59% for patients with cPCs ≥0.165% and 68% for patients with cPCs <0.165% (p=0.109) (**Figures 3A, B**).

There were 21 (14.5%) patients who were classified as R-ISS I, 97 (66.9%) as R-ISS II, and 27 (18.6%) as R-ISS III. Among the R-ISS I patients, there was no patient with progression and death. For the R-ISS II patients, they were further divided into two groups based on the presence or absence of cPCs ≥0.165%. The median PFS was 36 months in the group with cPCs ≥0.165% and was not reached in the group with cPCs <0.165% (p=0.082). The 3-year OS was 78% in the group with cPCs ≥0.165% and 90% in the group with cPCs <0.165% (p=0.206). Comparing patients with R-ISS III and R-ISS II with cPCs ≥0.165%, there were no significant differences in PFS (median PFS, 33 months vs. 36 months, p=0.659) or 3-year OS (58% vs. 78%, p=0.245) (**Figures 3C, D**).

## Discussion

In recent years, there has been growing interest in the prognostic significance of cPCs in plasma cell diseases such as MM, SMM, and MGUS (13–15). The presence of cPCs in the peripheral blood has been consistently associated with poor prognosis, both after treatment and during the relapsed phase (18). In this study, we analyzed the clinical data of 145 patients with NDMM and evaluated the prognostic value of cPCs. Based on our analysis, we identified a cPCs threshold of ≥0.165% as the best predictor of poor prognosis. Approximately 30% of the NDMM cases in the high cPCs group exhibited characteristics such as higher BMPC levels, high-risk cytogenetics, advanced ISS stage, lower platelet counts, and a higher proportion of renal insufficiency. These findings suggested that patients with a high proportion of cPCs have more significant bone marrow suppression, a higher tumor burden, and more pronounced organ involvement.

This study revealed that a higher ratio of cPCs is associated with a poor prognosis in MM, regardless of other factors such as ASCT and ISS stage. Among the high-cPCs group, cases that underwent ASCT exhibited a worse PFS and OS compared to those with lower cPCs levels. Furthermore, among patients who did not undergo ASCT, those with cPCs ≥0.165% had significantly shorter median PFS (14 months) and OS (20 months), which is lower than the reported 6-year OS for this patient population (19).

The study also indicated that in the subgroup of patients with standard-risk cytogenetics without either t(4;14), t(14,16), or del(17p), those with cPCs ≥0.165% had significantly worse OS and PFS compared to those with cPCs <0.165%. However, among patients with high-risk cytogenetics with either t(4;14), t(14,16), or del(17p), the survival outcomes of those with high cPCs were similar to those with lower cPCs levels. It is important to note that these findings may be influenced by factors such as a shorter follow-up period and a limited number of subjects, which could potentially impact the observed outcomes.

A recent study highlighted that in patients with R-ISS stage I and II MM, the presence of more than 5 cPCs/μL, as detected by MFC, was associated with a worse prognosis compared to R-ISS stage III cases according to the Mayo Clinic classification (20). This suggests that these patients should potentially be reclassified as R-ISS stage III to reflect their poor prognosis. Another study found that in the R-ISS II group, both OS and PFS rates were remarkable higher in patients without cPCs at diagnosis compared to that with ≥1 cPC (21). In our study, patients with high cPCs who had R-ISS stage II have similar survival outcomes to those of stage III patients with median PFS (33 vs. 36 months, p=0.659) and 3-year OS (78% vs. 58%, p=0.245). These findings are consistent with the Mayo Clinic’s observations, suggesting that the presence of ≥0.165% cPCs detected by MFC can potentially upstage a subset of NDMM patients within the R-ISS system. However, it is important to note that the number of patients with high levels of cPCs was small, and there were no cases with progression or death in the R-ISS stage I group, likely due to the limited sample size and short follow-up period. Future studies should include a larger sample size and longer follow-up time to further investigate these associations and provide more robust conclusions.

The high number of cPCs related to worse prognosis may be attributed to cytogenetic abnormalities. The Mayo Clinic study found that cases with cPCs are more prone to the occurrence of high-risk cytogenetics by FISH, and for such cytogenetic abnormalities, the frequency of t(4;14) and deletion 13q were higher in the subgroup with cPCs (22). Another study found that cases with cPCs ≥0.105% had a higher risk of harboring P53 deletion and more cases with high-risk genetic variations compared to those with cPCs <0.105% (23). In addition, patients with pPCL were found to exhibit higher cytogenetic changes than those with NDMM, including a correlation between higher cPCs and specific cytogenetic abnormalities such as t(11;14) and deletion 17p (24). In our study, we also found that patients in the cPCs ≥0.165% group had a higher prevalence of high-risk cytogenetic changes compared to those in the cPCs <0.165% group. However, the specific mechanisms for these relationships remain elusive. Paiva et al. (25) pointed out that cPCs express low levels of integrins (e.g., CD11a and CD49d) and adhesion molecules (e.g., CD33), which could contribute to the increase in invasion of cPCs. This could be another possible explanation for the association between high cPCs and adverse outcomes.

This study has several limitations that should be acknowledged. First, it was a retrospectively analysis, which may introduce inherent biases and limitations associated with retrospective studies. This study was conducted at a single center, and the threshold of ≥0.165% cPCs was mainly determined based on a single study that needs to be confirmed in conjunction with other studies. Third, the sample size in the research was limited, and follow-up period was relatively short, which could restrict the reference value of the obtained results. In future research, it is necessary to increase the sample size and extend the follow-up time so as to obtain more reliable results.

## Data availability statement

The original contributions presented in the study are included in the article/supplementary material. Further inquiries can be directed to the corresponding authors.

## Ethics statement

The studies involving humans were approved by The First Affiliated Hospital of Soochow University. The studies were conducted in accordance with the local legislation and institutional requirements. The participants provided their written informed consent to participate in this study.

## Author contributions

WY: Conceptualization, Data curation, Formal Analysis, Writing – original draft. HFY: Conceptualization, Data curation, Formal Analysis, Writing – original draft. HYY: Conceptualization, Data curation, Formal Analysis, Writing – original draft. JS: Investigation, Writing – review & editing. YZ: Investigation, Writing – review & editing. ZY: Investigation, Writing – review & editing. SY: Methodology, Writing – review & editing. XS: Methodology, Writing – review & editing. YY: Writing – review & editing, Project administration, Resources. JW: Writing – review & editing, Project administration, Resources. PW: Project administration, Resources, Writing – review & editing. YX: Project administration, Resources, Writing – review & editing. SJ: Writing – review & editing, Visualization. LY: Writing – review & editing, Validation. DW: Supervision, Validation, Visualization, Writing – review & editing. CF: Supervision, Validation, Visualization, Writing – review & editing.
